# The Effect of Openings’ Size and Location on Selected Dynamical Properties of Typical Wood Frame Walls

**DOI:** 10.3390/polym14030497

**Published:** 2022-01-26

**Authors:** Marcin Szczepanski, Ahmed Manguri, Najmadeen Saeed, Daniel Chuchala

**Affiliations:** 1 Faculty of Civil and Environmental Engineering, Gdansk University of Technology, 80-233 Gdansk, Poland; marcin.szczepanski@pg.edu.pl (M.S.); ahmed.manguri@pg.edu.pl (A.M.); 2 EkoTech Center, Gdańsk University of Technology, 80-233 Gdańsk, Poland; 3 Civil Engineering Department, University of Raparin, Ranya 46012, Kurdistan Region, Iraq; najmadeen_qasre@uor.edu.krd; 4 Civil Engineering Department, Tishk International University, Erbil 44001, Kurdistan Region, Iraq; 5 Faculty of Mechanical Engineering and Ship Technology, Gdansk University of Technology, 80-233 Gdansk, Poland

**Keywords:** wood-frame, OSB boards, composite materials in buildings, dynamic loads, natural frequency, openings

## Abstract

The wooden frame constructions are now popular in many developed countries of the world. Many of these locations where such buildings are constructed are exposed to seismic and other shocks which are generated by human activities. This paper discusses the effect of the size and location of openings in the wooden frame walls under dynamic loadings. Natural frequencies of such frames with and without openings have been determined. Three 14 m high walls with different widths, including 3, 6, and 12 m, have been considered. Dynamic analysis has been made using finite element method structural analysis software Dlubal RFEM 5.17. The results show that the effect of the size and location of the openings on the natural frequency is significant. Numerically speaking, the relative change of the natural frequencies of a wall without and with an opening in a specific place could be up to 30%. In addition, the change of the natural frequency for the location of the openings is more sensitive than that to the sizes. Furthermore, the appropriate sizes and locations of openings of the wooden frame walls have been suggested. The appropriate size and place were found to be small openings in the top of the walls.

## 1. Introduction

**Figure 1:**
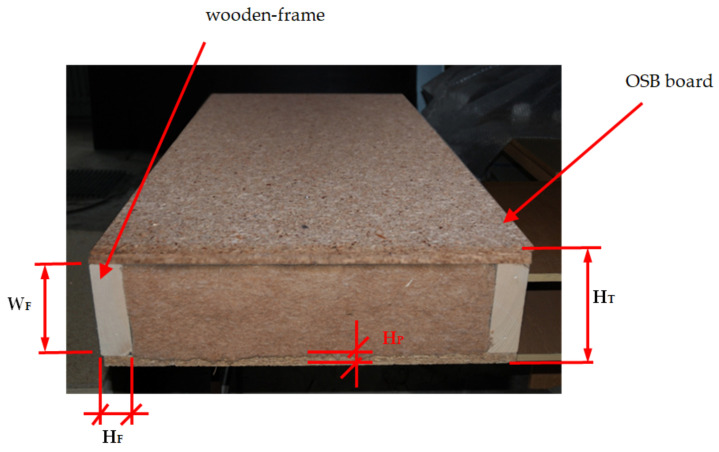
Example wall module for standard wooden-frame construction, where W_F_ is width of frame beam, H_F_ is height of frame beam, H_P_ is thickness of plate, H_T_ is thickness of wall.

The wooden frame constructions are now popular in developed countries such as USA, Canada, Australia, and many European countries. This is due to the very good mechanical properties of wood and wood-based materials, which are increasingly used in the building industry [1]. The environmental aspect is also important, as timber-frame houses have a significant impact on reducing CO_2_ emissions compared to concrete buildings [2]. Additionally, wooden structures perform better in certain climatic conditions [3]. Wood-frame wall structures consist of a frame made of sawn timber and an oriented standard boards (OSB) casing, with the center filled with thermal insulation material (Figure 1). The frames for walls are usually made of pine or spruce timber, which is properly sorted to meet the requirements for mechanical properties [4]. The boards surrounding the frame are most often OSB boards, which have good physical and mechanical properties [5,6]. However, other solutions are often proposed based on the use of wood waste or other biomasses [7,8,9]. The inside of the frame is filled with an insulating material that achieves various functions: thermal insulation [10], acoustic insulation [11,12], and structural reinforcement [13]. Insulation materials are often mineral wool [10], polyurethane foams (PU foams) [13,14,15], or other solutions based on waste from various biomasses [15,16,17].

In general, shear walls are the main elements of the timber structures that resist the lateral loads and earthquake excitation loads [18]. Different techniques have been implemented to evaluate and enhance the earthquake resistance of timber structures. X-Lam buildings’ ductile behavior has been measured under dynamic loads, and the buildings were modeled to perform non-linear time-history analyses for design purposes [19]. Furthermore, CLT (Cross Laminate Timber) wall panels have been tested to understand their behavior under a quasi-static lateral load [20]. Some of these studies have been conducted through numerical models.

Numerical modeling is considered an economical and easy to perform analysis rather than experimental works. Numerical calculations use finite element methods that give relatively accurate results [14]. Nowadays, the focus of the researchers on these models due to their efficiency, time, and cost-saving has been increased. Generally speaking, there are two types of wooden frame structures. The first one is prefabricated wooden frame construction, in which the structure is built by connecting colossal, prefabricated elements [21]. The second is a light frame construction system using many small and closely spaced members that can be assembled by nailing [22]. The latter will be considered in this study.

In order to improve the quality of occupants’ lives, engineers are trying to modernize the structures. One of such ways is adding additional openings or widening the current ones. The openings have a substantial impact on the structures’ dynamic behavior [23]. Since openings can significantly affect the structural properties of buildings, researchers studied this effect on overall stiffness of CLT structures [23] and shear properties of wooden walls [24,25]. The effect of size and shape of openings on the stiffness and shear behaviors of CLT walls has also been estimated [26]. The aim of this work is the analysis of natural frequencies of vibrations based on different sizes and locations of openings in wooden frame walls. Furthermore, the appropriate size and location of the openings in the wooden walls will be suggested. Since, in the modern life, the openings are performed in the walls for different purposes, they have negative impact on the structural performance if they are carried out in arbitrary places.

## 2. Methodology

In this section, the properties and dimensions of the walls and the openings are introduced.

### 2.1. Wall Geometries and Material Properties

Three walls with different width, 3 (W1), 6 (W2), and 12 m (W3), each have 14 m height and are used as numerical models in this research; these are typical dimensions of the prefabricated walls. The walls were modeled as connected panels with dimensions of 0.60 m length, a height of 1.20 m, and a thickness of 0.15 m. The panels were constructed as four connected wooden boards made of the coniferous timber, Scots Pine (*Pinus sylvestris* L.) of C18 strength classes [27], which create a frame covered on both sides with oriented standard boards (OSB3). The properties of the frame [28] and the sheathing [13,29], according to Eurocode 5 [30], have been tabulated in Table 1. To make modeling of the walls as real as possible, the support reactions were assumed to be hinged (i.e., the transitions were inhibited while the rotations were permitted).

### 2.2. Numerical Models of Wooden Walls

**Figure 2:**
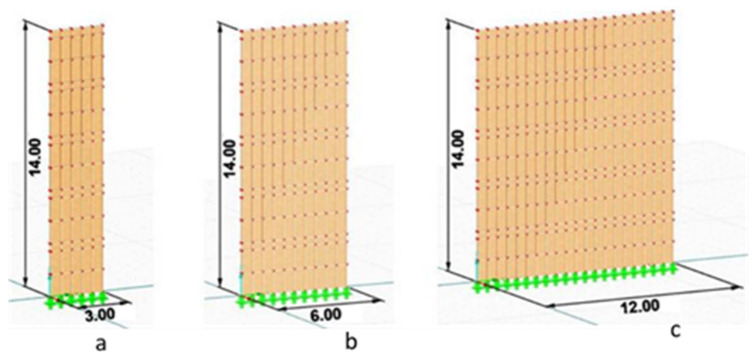
Dimensions of analyzed walls: (**a**) W1, (**b**) W2, (**c**) W3.

**Figure 3:**
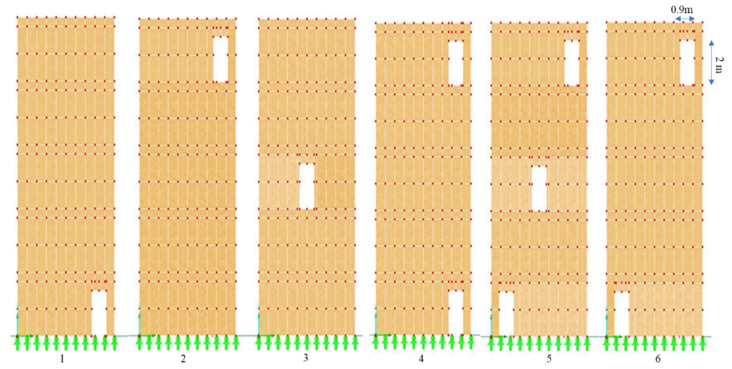
Location of openings on W2. 1–6 are the different positions of the openings with dimensions 0.9 × 2 m.

For each wall, several scenarios of openings in terms of location and size have been evaluated. Firstly, the three walls with 3, 6, and 12 m wide walls without openings have been tested (see Figure 2). Secondly, the openings assumed to be 0.9 × 2 m (Op1) in different locations on W1, W2, and W3. For W2, the illustration is given in Figure 3. Same locations allocated for 1.8 × 2 m (Op2) for W1, W2, and W3.

**Figure 4:**
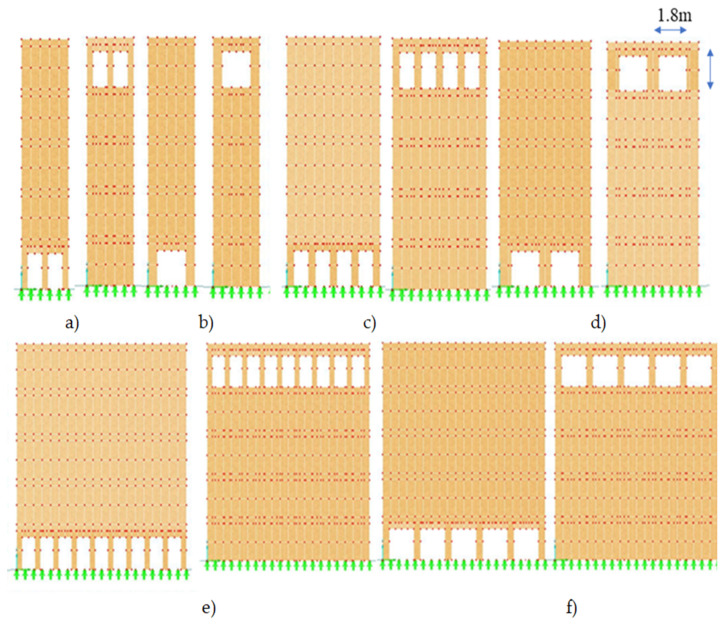
Location and size of horizontal system openings of three different widths of wooden frame walls. The walls 3 m wide (W1) with openings 0.9 × 2 m (Op1) (**a**) and openings 1.8 × 2 m (Op2) (**b**); the walls 6 m wide (W2) with openings 0.9 × 2 m (Op1) (**c**) and openings 1.8 × 2 m (Op2) (**d**); the walls 12 m wide (W3) with openings 0.9 × 2 m (Op1) (**e**) and openings 1.8 × 2 m (Op2) (**f**).

Another type of system opening is the horizontal arrangement in each story. It means that the holes were placed next to each other at the same distance. The distance between two adjacent openings is set to be different for the three various width walls. For W1, the distance between two Op1s is equal to 0.3 m and for Op2s is 0.6 m, whereas for W2, the two adjacent Op1s have the distance equal to 0.48 m and for two adjacent Op2s, their distance is 0.8 m. Furthermore, the distance between two Op1s and Op2s were set to be 0.39 and 0.5 m, respectively, for W3. Figure 4 shows the openings in top and bottom level of W1, W2, and W3.

**Figure 5:**
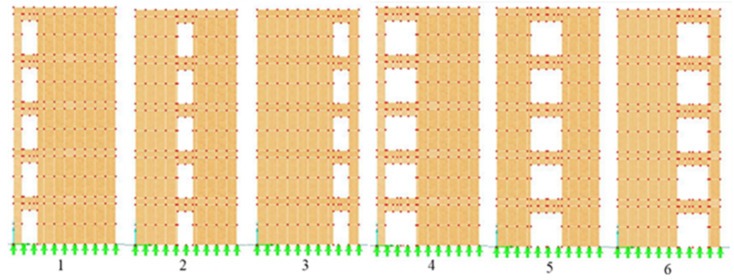
Locations of vertical opening system of W2. 1–3 are the different positions of the openings with dimensions 0.9 × 2 m. 4–6 are the different positions of the openings with dimensions 1.8 × 2 m.

For the vertical opening system, the openings were located one above another on every floor with prime displacement equal to 0.5 m from the left edge of the wall. Then, the edge distance was increased by 0.5 m in every next step until the holes approached the opposite edge of the wall. An example of this type for Op1 and Op2 for W2 is shown in Figure 5; the same scenarios have been performed for W1 and W3.

## 3. Modal Analysis

The analysis was made by the finite elements method in Dlubal RFEM 5.17 software (Dlubal Software GmbH, Tiefenbach, Germany). The analysis shows that the systems have three natural vibrations; this is due to the fact that these kinds of frequencies appear in real five floor buildings [31]. The influence of location and size of openings was estimated by calculating the relative change of natural frequencies using the equation below:

(1) $$d_{i}=\frac{f_{oi}-f_{i}}{f_{i}}*100,i=1:3$$

where $d_{i}$ is the relative change of ith natural frequency, $f_{i}$ is ith the natural frequency for the wall without openings, $f_{oi}$ is ith the natural frequency for wall with openings.

Table 2 shows the response of W1, W2, and W3 without openings to the dynamic loading. The data from the table declares that the lowest frequency appears from the first natural frequency (1st NF) for the three walls, while the middle size wall recorded the lowest 1st NF among all.

**Figure 6:**
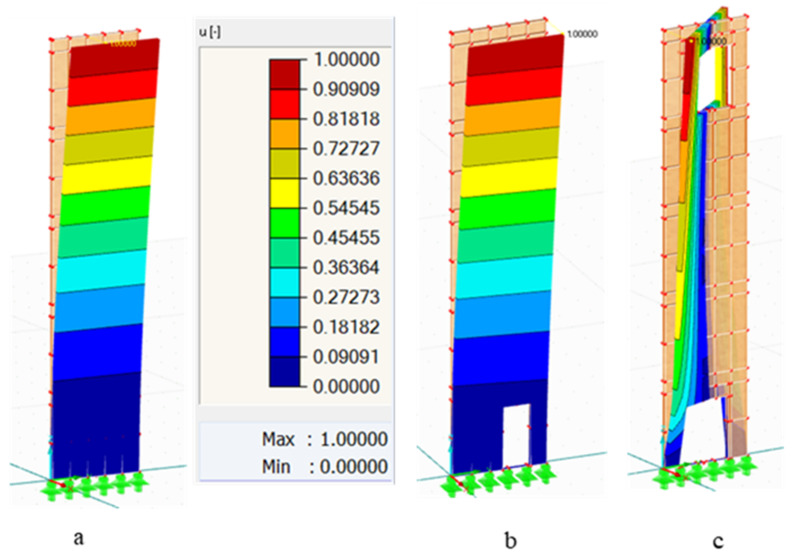
NF of W1: (**a**) 1st NF without openings, (**b**) 1st NF with Op1 at 1st position, and (**c**) 3rd NF with Op2 at 6th position.

In Figure 6, the responses of W1 without and with openings to dynamic loading have been shown; Figure 6a is the 1st form of vibration for the wall without openings. While Figure 6b exemplifies the 1st NF of the wall with Op1 in the right bottom of the wall, Figure 6c is the 3rd NF of the opened wall in the bottom and the top by 1.8 × 2 m. The analysis has been performed for the three walls with different sizes and locations of the openings; the results were tabulated below.

The three natural forms of vibration after applying the dynamic load for Op1 and Op2 in six different locations of three various width walls were presented in Table 3. It can be clearly seen that during the 1st and 2nd NF, the lowest value is recorded when the opening is in the right bottom of the walls, while the highest value is recorded for the three forms of NF when the opening is located in the top right of the walls. However, the situation was different regarding the lowest value for the 3rd NF; the smallest NF was obtained when there were three openings in the right diagonal. Numerically speaking, the highest and lowest values were obtained for W1 with Op2. The former recorded 4.048 Hz, which was obtained from the 3rd NF for Op2 in the right top, and the latter recorded 0.29 Hz, which was attained from the 1st NF for the same size of the opening, but its location was in the right bottom.

Table 4 shows the three forms of vibration for Op1 and Op2 in three different scenarios for the three size walls for the horizontal openings. It could be said that when the openings were in the top story, high natural frequencies were recorded, whereas when they were located in the bottom, low values of NF were obtained. To be more specific, the highest frequency was gained for the 3rd NF of the top Op1, while the smallest value was obtained for the 1st NF of the same opening size but in the bottom floor (see Figure 4).

Table 5 shows three forms of natural frequencies of the walls for the vertical system openings. The table demonstrates that the values of the 1st and 3rd NF almost remain unchanged by changing the size and location of the openings, while the 2nd NF became higher by decreasing the size of the wall and the openings. The table clearly shows that the highest frequency gained for W2 from the 3rd NF was when Op1 had 0.5 m distance from the left (see Figure 5(1)), while the smallest value found for the same size wall was from the 1st NF when Op2 was located 0.5 m away from the left (see Figure 5(4)).

## 4. Relative Change of Natural Frequencies

The relative changes have been derived between the natural frequencies of the walls with and without openings. Furthermore, the figures illustrate the relative change of three forms of the natural frequencies for different sizes of openings, namely Op1 and Op2 in different locations that are shown in Figure 3, Figure 4 and Figure 5. According to Equation (2), *f* is directly proportional with *K* and inversely proportional with *M*.; hence, when an opening is performed in a wall, *K* and *M* decrease. However, the diminishing of each depends on the size and the location of the opening. If *K*/*M* of the opened wall is greater than that of the non-opened wall, it means *f* is enhanced. Thus, the figures clearly illustrate the positivity and negativity of the relative change of the size and locations of openings of each wall size.

(2) $$f=\sqrt{\frac{K}{M}}$$

where *K* and *M* are the stiffness and the mass of the wall.

**Figure 7:**
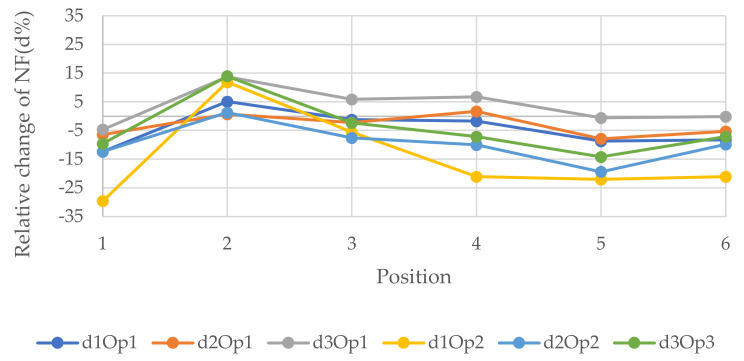
Relative changes of the natural frequencies for W1.

**Figure 8:**
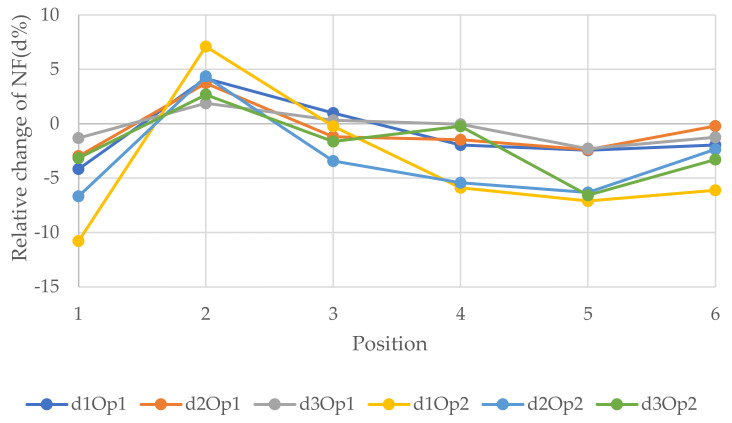
Relative changes of the natural frequencies for W2.

**Figure 9:**
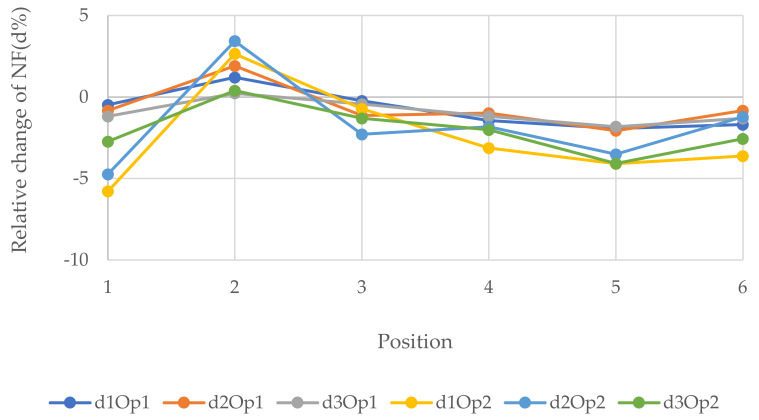
Relative changes of the natural frequencies for W3.

Figure 7, Figure 8 and Figure 9 represent the relative changes (d1, d2, and d3) of the natural frequencies of W1, W2, and W3, respectively. One can see from Figure 7, the maximum relative changes obtained for Op1 in the 3rd NF and Op2 in the 1st and 3rd NF in location 2, shown in Figure 3, that the relative change was about 15%. On the other hand, the least relative change gained for Op2 in the 1st NF in location 1, shown in Figure 3, was 30%. In general, the charts in the three figures show that the relative changes of all three forms of NF for both opening sizes were positive when the opening was located in the 2nd position, whereas the relative changes in the majority of the other cases were negative when the openings were located other than in position 2, which is the top right of the wall.

**Figure 10:**
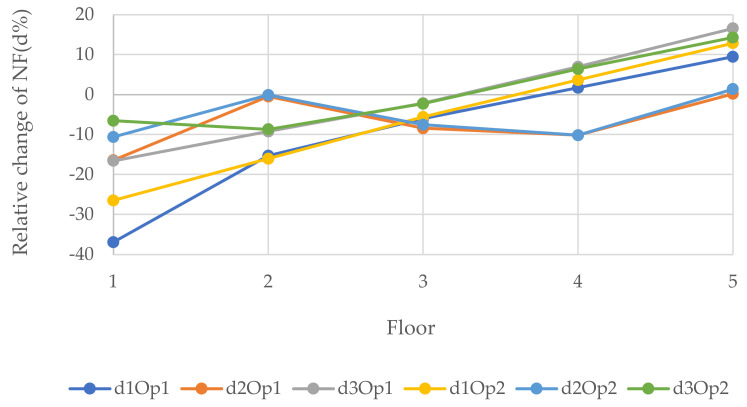
Relative changes of the natural frequencies for W1.

**Figure 11:**
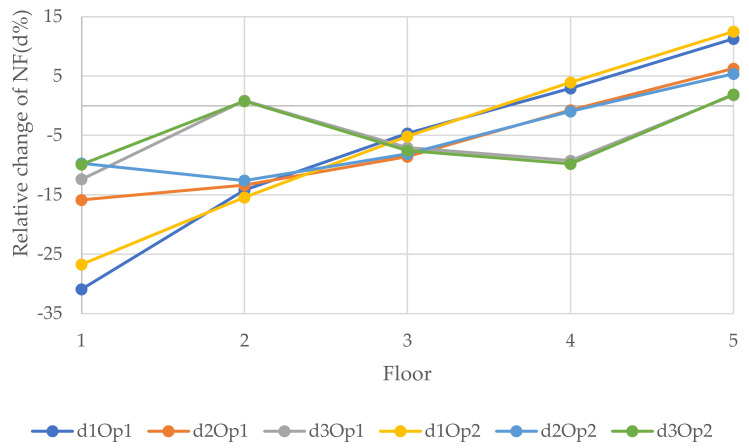
Relative changes of the natural frequencies for W2.

**Figure 12:**
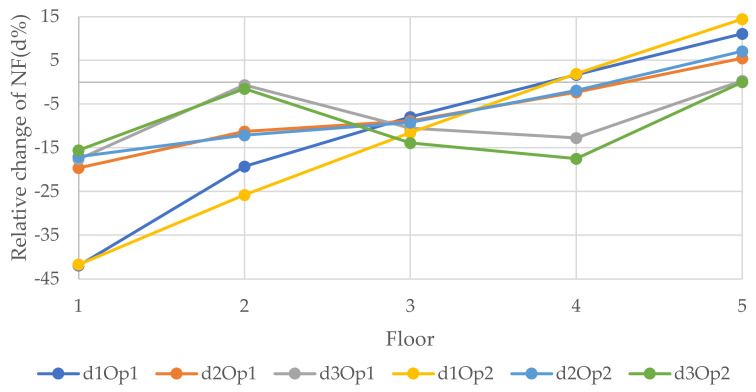
Relative changes of the natural frequencies for W3.

Figure 10, Figure 11 and Figure 12 epitomize the relative changes (d1, d2, and d3) of the natural frequencies of the walls with the width 3, 6, and 12 m, respectively. As can be seen, the most considerable positive change of the 3rd natural frequency was for the 3 m wide wall with 1.8 × 2 m openings located in position 5, which is the fifth floor, and it was just over 15%. The reason for this could be that when the openings are performed at the bottom of the wall, the structure will be vulnerable. In contrast, when the openings are operated at the top level, the dynamic load will be least effective on the structure. Usually, for W2 and W3, the changes were similar for a particular position. However, there was a rapid increase in relative change due to a change in opening location in the 1st NF for both size openings for all size walls. Generally, the positive relative changes gained when the opening were located on the top floor, in contrast to the negative relative changes gained when the openings were on the 1st floor.

**Figure 13:**
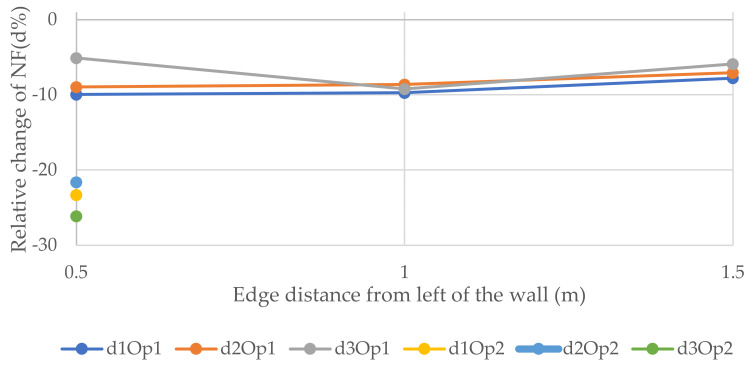
Relative changes of the natural frequencies for W1.

**Figure 14:**
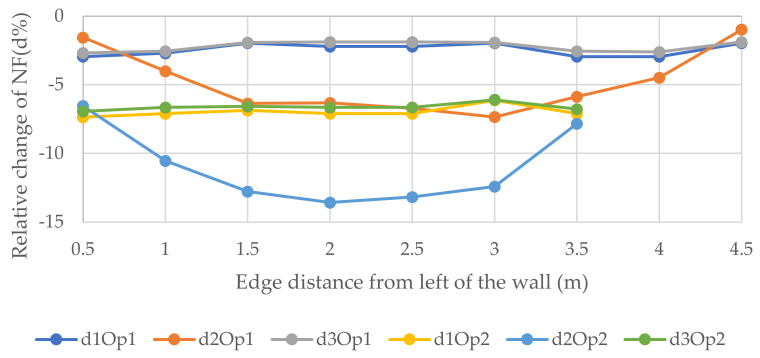
Relative changes of the natural frequencies for W2.

**Figure 15:**
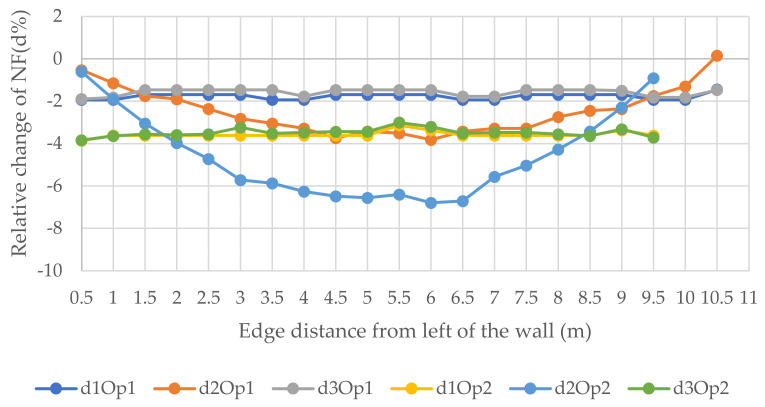
Relative changes of the natural frequencies for W3.

Figure 13, Figure 14 and Figure 15 show the relative changes (d1, d2, and d3) of the natural frequencies of the walls with the change of the distance of the vertical openings to the left edge of the walls (see Figure 6). As can be seen from the figures, for the 1st and 3rd relative NF for both sizes of openings for 6 and 12 m walls, the lines remained almost flat with the change of the distance of the openings with the left edge of the wall. However, the 2nd RNF has the highest value when the opening is at the edges, and it declines when the opening is close to the middle of the wall and records the lowest value when the opening is at the middle of the wall. Although, for the 3 m wide wall the situation is different; for the 0.9 × 2 m opening, change is decreasing from the beginning, and for the 1.8 × 2 m opening, there is only one value because the hole consists of almost the whole width of the wall and, in this position, the relative change of the 2nd, 1st, and 3rd forms recorded the lowest values, −21.6, −23.3, and −26.1%, respectively. It could be said that the openings in the middle of the wall for the vertical system are considered to be vulnerable.

## 5. Conclusions

In this paper, three walls with different widths without and with two different sizes of openings in several locations have been tested to monitor the change in their natural frequencies under dynamic loads. It has been concluded that:

- Designing new openings with different sizes and locations can significantly affect the change of the values of the natural frequencies.
  ○ Small openings give better results than the big openings.
  ○ Vertical openings are the most unfavorable system of openings.
- For the 1st six positions of openings, the highest and lowest natural frequencies were recorded when the openings were located in the top right and bottom right of the walls, respectively. The relative change of the natural frequencies also gave the same outcomes.
- For the horizontal system of openings, the most suitable position for performing the openings is the top floor, while the 1st floor is considered as the worst scenario.
- Regarding the vertical openings, the appropriate case is when the openings are located at the edges, while the inappropriate case scenario has openings in the middle wall.
- According to the research, the appropriate size and location of the openings mitigate the impact of the seismic excitations to the timber frame walls.
- Further study can be conducted through dealing with the effect of the openings on a whole structure.

## Figures and Tables

**Table 1:** Material properties of different elements used in the wooden frame wall [4,5].

Element	Material	Density (kg/m^3^)	Elasticity Modulus (GPa)
Frame	Pine Wood of class C18	430	9.0-along fibers 0.3-across fibers
Sheathing	OSB 3	713.8	4.93-along fibers 1.98-across fibers

**Table 2:** Tree NF forms of the walls without openings.

Wall Width (m)	Frequency (Hz)		
	Form 1	Form 2	Form 3
3	0.412	2.525	3.552
6	0.408	2.248	2.496
12	0.415	1.311	2.530

**Table 3:** NF for W1, W2, and W3 with Op1 and Op2 shown in Figure 3.

Position	Frequency (Hz)																	
	3 m Wall						6 m Wall						12 m Wall					
	Opening Sizes																	
	0.9 × 2 m			1.8 × 2 m			0.9 × 2 m			1.8 × 2 m			0.9 × 2 m			1.8 × 2 m		
	Forms																	
	1	2	3	1	2	3	1	2	3	1	2	3	1	2	3	1	2	3
1	0.361	2.363	3.387	0.290	2.213	3.211	0.391	2.181	2.463	0.364	2.098	2.417	0.413	1.301	2.501	0.391	1.249	2.461
2	0.433	2.543	4.039	0.461	2.557	4.048	0.425	2.332	2.543	0.437	2.346	2.563	0.420	1.336	2.536	0.426	1.356	2.540
3	0.407	2.468	3.760	0.389	2.334	3.467	0.412	2.221	2.504	0.407	2.171	2.455	0.414	1.296	2.519	0.412	1.281	2.497
4	0.405	2.567	3.792	0.325	2.271	3.297	0.4	2.215	2.495	0.384	2.126	2.49	0.409	1.298	2.500	0.402	1.287	2.479
5	0.376	2.326	3.531	0.321	2.035	3.048	0.398	2.194	2.439	0.379	2.106	2.332	0.407	1.284	2.484	0.398	1.265	2.427
6	0.378	2.39	3.547	0.325	2.276	3.297	0.4	2.243	2.465	0.383	2.195	2.414	0.408	1.300	2.497	0.400	1.295	2.465

**Table 4:** NF for W1, W2, and W3 with Op1 and Op2 of the horizontal system shown in Figure 4.

Floor	Frequency (Hz)																	
	3 m Wall						6 m Wall						12 m Wall					
	Opening Sizes																	
	0.9 × 2 m			1.8 × 2 m			0.9 × 2 m			1.8 × 2 m			0.9 × 2 m			1.8 × 2 m		
	Forms																	
	1	2	3	1	2	3	1	2	3	1	2	3	1	2	3	1	2	3
1	0.260	2.111	2.964	0.303	2.258	3.319	0.282	1.892	2.187	0.299	2.030	2.250	0.241	1.054	2.085	0.242	1.088	2.136
2	0.349	2.513	3.225	0.346	2.522	3.244	0.350	1.947	2.518	0.345	1.965	2.516	0.335	1.163	2.514	0.308	1.152	2.493
3	0.387	2.312	3.476	0.389	2.335	3.470	0.389	2.055	2.320	0.387	2.066	2.307	0.382	1.195	2.264	0.367	1.190	2.178
4	0.419	2.268	3.799	0.427	2.269	3.779	0.420	2.231	2.265	0.424	2.227	2.252	0.422	1.281	2.207	0.423	1.286	2.088
5	0.451	2.530	4.140	0.465	2.560	4.059	0.454	2.389	2.541	0.459	2.369	2.544	0.461	1.383	2.539	0.475	1.404	2.531

**Table 5:** NF for W1, W2, and W3 with Op1 and Op2 of the vertical system shown in Figure 5.

Edge Distance (m)	Frequency (Hz)																	
	3 m Wall						6 m Wall						12 m Wall					
							Opening Size											
	0.9 × 2 m			1.8 × 2 m			0.9 × 2 m			1.8 × 2 m			0.9 × 2m			1.8 × 2 m		
	Forms																	
	1	2	3	1	2	3	1	2	3	1	2	3	1	2	3	1	2	3
0.5	0.371	2.299	3.371	0.316	1.979	2.624	0.396	2.213	2.429	0.378	2.101	2.323	0.407	1.304	2.482	0.399	1.303	2.433
1	0.372	2.307	3.225	-	-	-	0.397	2.158	2.432	0.379	2.011	2.330	0.407	1.296	2.484	0.400	1.286	2.438
1.5	0.380	2.347	3.342	-	-	-	0.400	2.105	2.448	0.380	1.961	2.332	0.408	1.288	2.493	0.400	1.271	2.440
2	-	-	-	-	-	-	0.399	2.106	2.449	0.379	1.943	2.330	0.408	1.286	2.493	0.400	1.259	2.439
2.5	-	-	-	-	-	-	0.399	2.097	2.449	0.379	1.952	2.330	0.408	1.280	2.493	0.400	1.249	2.440
3	-	-	-	-	-	-	0.400	2.083	2.448	0.383	1.969	2.344	0.408	1.274	2.493	0.400	1.236	2.448
3.5	-	-	-	-	-	-	0.396	2.116	2.432	0.379	2.072	2.327	0.407	1.271	2.493	0.400	1.234	2.441
4	-	-	-	-	-	-	0.396	2.147	2.431	-	-	-	0.407	1.268	2.485	0.400	1.229	2.442
4.5	-	-	-	-	-	-	0.400	2.226	2.449	-	-	-	0.408	1.262	2.493	0.400	1.226	2.443
5	-	-	-	-	-	-	-	-	-	-	-	-	0.408	1.266	2.493	0.400	1.225	2.443
5.5	-	-	-	-	-	-	-	-	-	-	-	-	0.408	1.265	2.493	0.402	1.227	2.454
6	-	-	-	-	-	-	-	-	-	-	-	-	0.408	1.261	2.493	0.401	1.222	2.449
6.5	-	-	-	-	-	-	-	-	-	-	-	-	0.407	1.266	2.485	0.400	1.223	2.441
7	-	-	-	-	-	-	-	-	-	-	-	-	0.407	1.268	2.485	0.400	1.238	2.442
7.5	-	-	-	-	-	-	-	-	-	-	-	-	0.408	1.268	2.493	0.400	1.245	2.442
8	-	-	-	-	-	-	-	-	-	-	-	-	0.408	1.275	2.493	0.400	1.255	2.440
8.5	-	-	-	-	-	-	-	-	-	-	-	-	0.408	1.279	2.493	0.400	1.266	2.438
9	-	-	-	-	-	-	-	-	-	-	-	-	0.408	1.280	2.492	0.401	1.281	2.446
9.5	-	-	-	-	-	-	-	-	-	-	-	-	0.407	1.288	2.484	0.400	1.299	2.436
10	-	-	-	-	-	-	-	-	-	-	-	-	0.407	1.294	2.484	-	-	-
10.5	-	-	-	-	-	-	-	-	-	-	-	-	0.409	1.313	2.493	-	-	-

## Data Availability

The data presented in this study are available upon request from the corresponding author.
